# Shot Peening Effect on Sliding Wear in 0.9% NaCl of Additively Manufactured 17-4PH Steel

**DOI:** 10.3390/ma17061383

**Published:** 2024-03-18

**Authors:** Mariusz Walczak, Aleksander Świetlicki, Mirosław Szala, Marcin Turek, Dariusz Chocyk

**Affiliations:** 1 Department of Materials Engineering, Faculty of Mechanical Engineering, Lublin University of Technology, Nadbystrzycka 36, 20-618 Lublin, Poland; aleksander.swietlicki@pollub.edu.pl; 2 Institute of Physics, Maria Curie-Sklodowska University in Lublin, pl. M. Curie-Sklodowskiej 1, 20-031 Lublin, Poland; mturek@kft.umcs.lublin.pl; 3 Department of Applied Physics, Faculty of Mechanical Engineering, Lublin University of Technology, Nadbystrzycka 36, 20-618 Lublin, Poland; d.chocyk@pollub.pl

**Keywords:** additive manufacturing, DMLS, 17-4PH, AISI 630, shot peening, wear, tribology

## Abstract

The growing demand for modern steels showing corrosion and tribological resistance has led to their increased use in the production of medical devices. This study analyzed the effect of shot peening on wear resistance in 0.9% NaCl solution of 17-4PH steel produced by direct laser metal sintering (DMLS) technology. The study’s novelty relies on revealing the effect of shot peening (SP) surface treatment on the wet sliding wear resistance of 17-4PH steel produced with DMLS. Moreover, in the context of 17-4PH steel application for medical devices, the 0.9% NaCl tribological environment were selected, and SP processes were conducted using steel CrNi shot and ceramic (ZrO_2_) beads. The up-to-date scientific literature has not identified these gaps in the research. DMLS technology makes it possible to obtain products with complex architectures, but it also faces various challenges, including imperfections in the surface layer of products due to the use of 3D printing technology itself. The chemical and phase composition of the materials obtained, Vickers hardness, surface roughness, and microscopic and SEM imaging were investigated. Tribological tests were carried out using the ball-on-disc method, and the surfaces that showed traces of abrasion to identify wear mechanisms were subjected to SEM analysis. The XRD phase analysis indicates that austenite and martensite were found in the post-production state, while a higher martensitic phase content was found in peened samples due to phase transformations. The surface hardness of the peened samples increased by more than double, and the post-treatment roughness increased by 12.8% after peening CrNi steels and decreased by 7.8% after peening ZrO_2_ relative to the reference surfaces. Roughness has an identifiable effect on sliding wear resistance. Higher roughness promotes material loss. After the SP process, the coefficient of friction increased by 15.5% and 20.7%, while the wear factor (*K*) decreased by 25.9% and 32.7% for the samples peened with CrNi steels and ZrO_2_, respectively. Abrasive and adhesive mechanisms were dominant, featured with slight fatigue. The investigation showed a positive effect of SP on the tribological properties of DMSL 17-4PH.

## 1. Introduction

Steel 17-4PH, alternatively termed as AISI 630 (1.4542), is designed for precipitation hardening, which makes it possible to improve its strength [1]. This grade of steel is characterized by good corrosion resistance and high strength. When produced by conventional methods such as casting, it shows a martensitic structure, while after additive manufacturing, it is austenitic–martensitic steel, often containing ferrite [2]. The final microstructure depends on the composition of the steel powder as well as the manufacturing condition and parameters; thus, it is hard to predict the final martensite-to-austenite ratio [3]. Studies have shown that additively manufactured steel has similar and sometimes even higher mechanical properties than the steel produced by conventional methods [4]. Additive technologies allow the production of structures that are often impossible to produce by traditional methods and, despite the high cost, are cost-effective for a small batch or unit production [5]. According to Laleh et al. [6], the metal parts built directly from AM metal are often not ready for use in the as-built state, as their properties may not be optimized for their application, and the processing steps of final AM metals can account for about 27% of any costs. Despite its many advantages, the DMLS process encounters some imperfections related to the state of the surface layer of the products, among others. Even considering the optimal parameters of the printing technology recommended by manufacturers of metal powder laser sintering systems, the surface layer of products may contain unmelted grains of metal powder or pores, which are formed due to the collapse of the welding pool [7]. The above-mentioned surface imperfections can reduce the performance of such products. Most damage to the machine parts (stress cracks and abrasive or corrosive wear) originates from the defects occurring precisely in the surface layer. Research [8,9] on alloys dedicated to medical applications indicates that favorable surface layer properties can be obtained by shot peening treatment. The effect of SP treatment of 17-4PH steels from additive technologies on tribological characteristics has not yet sufficiently been explored. Most researchers focus on heat treatment as a method that is claimed to improve the properties of the steel [10]. As studies have shown, it does have a significant effect. However, the properties of 17-4PH steel can also be significantly improved by SP [11]. This is sometimes a highly desirable treatment that allows hardening of selected outer surfaces, leaving a ductile and elastic core. SP is a peening process that uses the kinetic energy of the shots to deform the treated surface plastically, usually imparted to them by a jet of compressed air [12]. There are also other methods such as ultrasonic impact peening, laser shot peening or cavitation peening [13]. The mechanism of strengthening during the SP process increases the number of dislocations in the near-surface layer, grain fragmentation occurs, residual compressive stresses are introduced and dislocation density is increased [14]. This can potentially result in reduced roughness, increased hardness [15] and improved fatigue strength [16]. The SP process also reduces structural defects such as cracks, voids and gas pores [17]. These properties, as well as easy implementation, make it a highly effective tool for improving resistance to tribological and fatigue processes [18]. The use of additive methods makes it possible to print a metal on another dissimilar metal, so more and more research is also focusing on multilayer combinations of dissimilar metals [19]. Such methods have also been applied to 17-4PH steel [20]. The SP process can be used to expand the ability to produce materials in this range. 17-4PH steel is used to produce biomedical devices such as knee-replacement surgery, surgical forceps, retractor blades and rings set used in a spinal surgery procedure, etc. [21]. Therefore, bearing in mind the above-mentioned areas of application, among others, resistance to abrasive wear in the environment of body fluids seems to be crucial. The corrosion resistance itself has already been studied by the authors of this paper, where the results were reported in several studies [22,23]. At that time, rather promising results were obtained for CrNi, ZrO_2_ and glass shot surfaces with regard to corrosion behavior. Very few researchers have attempted to describe the abrasive wear resistance of 17-4PH steel, and especially when it was additively manufactured. Most of the research in this field is conducted under ball-on-disc conditions [24,25,26]. Esfandiari and Dong [25] studied the effect of the wear resistance of heat-treated 17-4PH steel produced by conventional production (forming), which was additionally subjected to plasma nitrating. A ball-on-disc friction pair system was used in combination with dry sliding conditions combined with 3.5% NaCl solution. Abrasive and adhesive wear were identified for the non-nitrided samples. In contrast, the non-nitrided surfaces were worn significantly milder and characterized by micro-abrasion and oxidation wear. 

Esfandiari and Dong [25] studied the effect of wear resistance of heat-treated 17-4PH steel produced by conventional production (forming), which was additionally subjected to plasma nitrating. A ball-on-disc friction pair system was used in combination with dry sliding conditions combined with 3.5% NaCl solution. Abrasive and adhesive wear were identified for the non-nitrided samples. In contrast, the non-nitrided surfaces were worn significantly milder and characterized by micro-abrasion and oxidation wear. 

Sanjeev et al. [27] compared laser-based powder bed fusion (LB-PBF) 17-4PH steel with the wrought one. Wear performance was investigated using ball-on-disc method in dry sliding conditions with the load of 10 N and 30 N as well as in the lubricated condition (specimens were submerged in oil). It was noted that mostly abrasive mechanisms occurred. Lower wear rates were observed for LB-PBF in dry sliding conditions when compared to the wrought material. The LB-PBF 17-4PH SS, on the other hand, exhibited greater wear compared to the wrought 17-4PH SS in lubricated condition. This behavior was explained by the thinner lubricant layer formed on the surface as a result of the greater surface roughness of the LB-PBF samples. Adhesion was the dominant wear mechanism in the dry condition, while abrasion and surface fatigue were the wear mechanisms in the lubricated condition, regardless of the technology in which the steel samples were manufactured. The papers [27,28] pointed out the poor tribological properties of 17-4PH steel for forming (due to its lower surface hardness) compared to typical high-carbon bearing steels. On the other hand, an earlier study by the authors [23] indicates that surface hardness can be improved by shot peening treatment, which translates into higher resistance of DMLS 17-4PH under technically dry friction conditions. Not only can the surface layer condition and the environment influence tribological behavior, but the key ones according to Mahesh et al. [28] are three factors: normal load, sliding distances and sliding velocity. They demonstrated, that load is a more dominant factor relative to the sliding distance and sliding velocity in affecting the wear volume loss and specific wear rate.

As far as the authors’ knowledge goes, no description of the tribological behavior in 0.9% NaCl solution of shot peened 17-4PH stainless steel grade produced with DMLS technology is presented in the literature, especially in the context of its uses as medical devices. Above that, most of the research has been conducted in the heat-treated state or for steel produced by traditional methods. Given the gap in this field, the authors have undertaken this study to investigate DMLS 17-4PH steel wear resistance in 0.9% NaCl solution. Additionally, the novelty of this work is the use of steel directly after DMLS fabrication and the use of the SP process using steel CrNi shot and ceramic (ZrO_2_) beads to improve its tribological properties under environmental conditions.

## 2. Materials and Methods

### 2.1. Direct Laser Metal Sintering (DMLS) and Shot Peening of 17-4PH Samples

**Figure 1:**
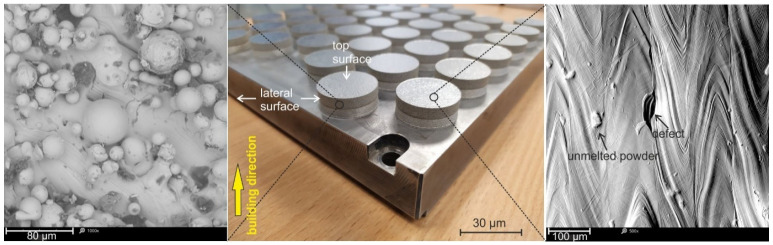
Lateral and top surface characteristic for additively manufactured 17-4PH steel, SEM.

Material manufacturing was performed with DMLS technology, and 6 samples were made using an EOS M280 printer (EOS GmbH Krailling, Germany). GP1 gas-atomized powder by EOS GmbH (Krailling, Germany) was also used for fabrication with the following process parameters: the laser power was 200 W, the thickness of the sinter layer was 0.02 mm, the laser spot size was 0.1 mm and the scan speed 1000 mm/s. Following Equation (1), shown in [29], the calculated energy density for this research was 100 J/mm^2^:

(1) $$E=\frac{P}{Vth},$$

where the symbols are as follows: energy input per volume (*E*), energy density (J/mm^2^), *P*, laser power (W), *V*, scanning, speed (mm/s), *t*, layer thickness (mm), *h*, hatch spacing (mm). Nitrogen was used as a shielding gas during fabrication. The obtained specimens were in the shape of disks, with a diameter of 30 mm and a height of 6 mm. A parallel scan strategy with an alternating scan direction was adopted, and for the successive layers, the scanning direction was rotated at a 90° angle. The fabricated specimens were built horizontally orientated. The as-built parts were subjected to a stress-relieving annealing at 650 °C for 1 h in an argon atmosphere (Ar). The heat treatment process was carried out for the matrix together with the specimens in a Nabertherm GmbH (Lilienthal, Germany) model N41/H chamber furnace dedicated to annealing after sintering. The orientation of the print can affect the microstructure and mechanical properties of fabricated components of 17-4PH steel [29,30]. Therefore, in our case, the samples were fabricated in repeatable conditions, as shown in Figure 1. After manufacture, the test material was cleaned in an ultrasonic cleaner and then air dried with compressed air. Although the disks were printed according to the optimal parameters recommended by the manufacturer EOS, they bore surface defects typical for this additive method. Figure 1 shows the design of the resulting discs, highlighting the top and lateral surfaces. SEM images revealed the minimal presence of unmelted powder particles along with satellites and impurities on the lateral surface. The top surface revealed dimple defects (which are formed by the welding pool’s collapse) and not entirely melted powder particles along the splashes. Half-overlapping laser paths and scales resulting from the cooling of the weld pool were also revealed. Therefore, such surface characteristics of the obtained specimens further justify the need for shot peening treatment, which is a commonly applied procedure for additively manufactured components [31]. 

**Figure 2:**
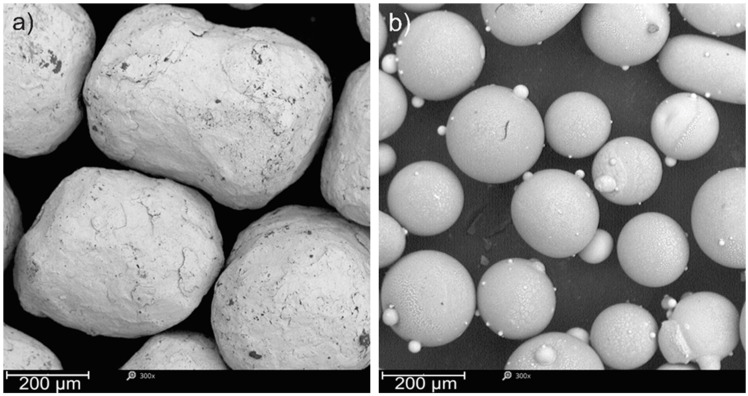
Shot particle morphology: (**a**) ZrO_2_ beads and (**b**) CrNi steel shots.

Following the fabrication of the specimens, the peening process with ceramic and steel balls shown in Figure 2 and Table 1 was carried out at constant parameters. Pressure of 0.4 MPa, a peening time of 60 s and a surface nozzle distance of 20 mm were applied. SP was performed on the top surface of the samples (top surface) using a Peenmatic micro 750S (IEPCO, Leuggern, Switzerland). Figure 1 shows the morphologies of the powders used in the SP process. The powders (Kuhmichel Abrasiv GmbH, Ratingen, Germany) used were characterized by an average grain size of 400–900 μm for CrNi shots, while the average size of ceramic particles was about 125–250 μm. The ceramic particles were mainly characterized by a spherical shape with satellites, while the CrNi shots particles were characterized by an irregular shape but close to a spherical one.

### 2.2. Characterization

The chemical composition was investigated using the Bruker (Billerica, MA, USA) Magellan Q8 spark emission spectrometer. Five sparks burn-throughs were made for each sample to calculate the mean values. The chemical composition testing was performed in order to confirm that the used materials are within the specification of the manufacturer as well as ASTM A564 and ISO EN10088-1 standards [32,33].

In order to investigate the microstructural characteristics, the metallographic specimens were prepared. Specimen preparation was performed by cutting the discs after peening and preparing the metallographic specimens that were flooded with epoxy resin. The specimens were ground against the papers ranging from #600, #800, #1200, #1800 and #2200 grit and then polished with a 3 µm diamond suspension. Etching was carried out in Kalling’s 1 and Marble’s reagent, after which the samples were polished again. Reagent selection was based on ASTM E407-07(2015) [34]. Metallographic specimens were examined using a Nikon (Irvine, CA, USA) MA200 optical microscope at ×200 magnification [22]. The surface texture morphology after shot peening tests was evaluated using a Phenom ProX scanning electron microscope (Phenom World, Waltham, MA, USA) with EDS at 500× magnification using topographic mode.

The phase composition was investigated in room temperature using a high-resolution X-ray diffractometer (XRD, Empyrean, Panalytical, Minato City, Tokyo) with Cu K-α radiation and Ni-filter with a generator voltage of 40 kV and a current of 30 mA. The specimen parameters were determined using the High Score Plus software package (version 3.0e, Malvern Panalitical, Malvern, UK). A proportional detector was used to detect radiation. The specimens were measured in the Bragg–Brentano geometry over a range 2θ = 30° to 100°, with step size of 0.01° and counting time of 6 s per data point applied. The fixed divergence slit of 1/4° was used together with a beam mask of 5 mm. The crystalline phase in the samples was identified using the High Score Plus software package with the Crystallography Open Database.

To determine the effect of the shot peening process on roughness, the roughness tests were conducted using a Dektak 150 contact profilometer (Veeco Instruments, Plainview, NY, USA). Measurements were taken using a stylus with a rounding radius of 2 µm at a measuring length of 5 mm and under a load of 3 mg; twelve measurements were taken for each sample at randomly selected locations. The average value was calculated from 12 measurements. To account for the effect of texture, 6 horizontal and 6 perpendicular measurements were taken on the scanning direction of the laser beam.

Hardness measurements of the modified surfaces were taken at a load of 300 gf–2.942 N (HV0.3, respectively); the dwell time was set at 15 s; and a Vickers’s FM-700 micro-hardness tester with an ARS 900 automatic system (Future-Tech Corp., Kawasaki, Japan) was used. Ten indentations were made for each group of specimens at randomly selected locations.

Wear tests were performed on a ball-on-disc tribotester (CSM Instruments, Yokohama, Switzerland) in a 0.9% NaCl solution (100 mL volume) at 22 °C. Balls made of Al_2_O_3_ (hardness 1680 HV0.5) with a diameter of 6 mm were used as a counterbody. Three repetitions were performed, and the tests were carried out under a load of 25 N with a linear speed of 1.88 cm/s at a radius of 3 mm. The total test distance was 100 m, during which the change in the coefficient of friction was recorded. The degree of wear was determined based on the wear coefficient *K*:

(2) $$K=\frac{Wear\;volume}{Applied\;force\;\times \;sliding\;distance}\left[{mm}^{3}N^{-1}m^{-1}\right]$$

The wear volume was calculated according to [27] by integrating the area across the wear track profile (using the 12 measurements) and then multiplying by the circumference length of the track. The surface of the wear tracks of the tested materials after tribological tests was evaluated using a Phenom ProX scanning electron microscope.

## 3. Results

### 3.1. Structure Analysis

High-precision measuring instruments enable one to establish chemical composition with sufficient accuracy. The chemical composition according to ASTM A564, EN10088-1 and the test results is given Table 2. The chemical composition test revealed the chemical composition of 17-4PH grade steel in accordance with the manufacturer declaration. In accordance with ASTM A564 and EN10088-1 standards, the tested 17-4PH steel alloy met the composition specifications quoted in the previously mentioned standards. In a previous study [22], the powder composition was similar to that obtained in the final element and similar to that obtained in this study. However, the obtained chemical composition results differed slightly when the C and Cu content was compared to the study on GP1 powder reuse [35]. 

**Figure 3:**
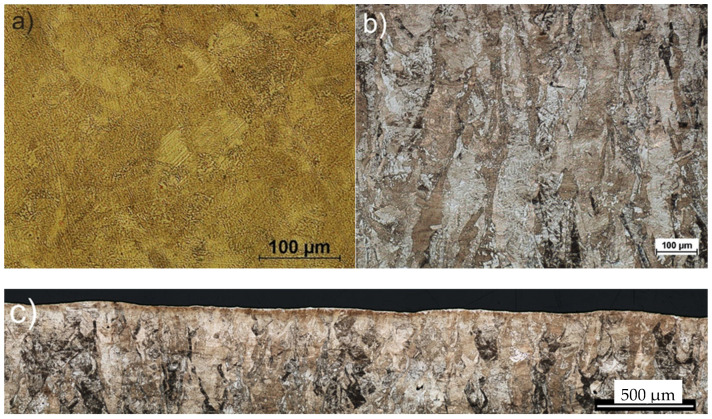
Microstructure of additively manufactured 17-4PH steel discs observed at (**a**) top surface and (**b**,**c**) lateral surface of a sample.

The microstructures shown in Figure 3 revealed a fine-grained austenitic–martensitic structure (Figure 3a) and overlapping fish scales (distinct melt pools—Figure 3c), with columnar grains molded in accordance with the building direction (Figure 3b). According to Clare et al. [36], their austenite grains overlapped, while the martensitic phase from this perspective was more irregular, with grains arranged both in parallel and transversely. However, the perpendicular cross-section to the building direction also suggests that it perhaps may have been the laser paths.

**Figure 4:**
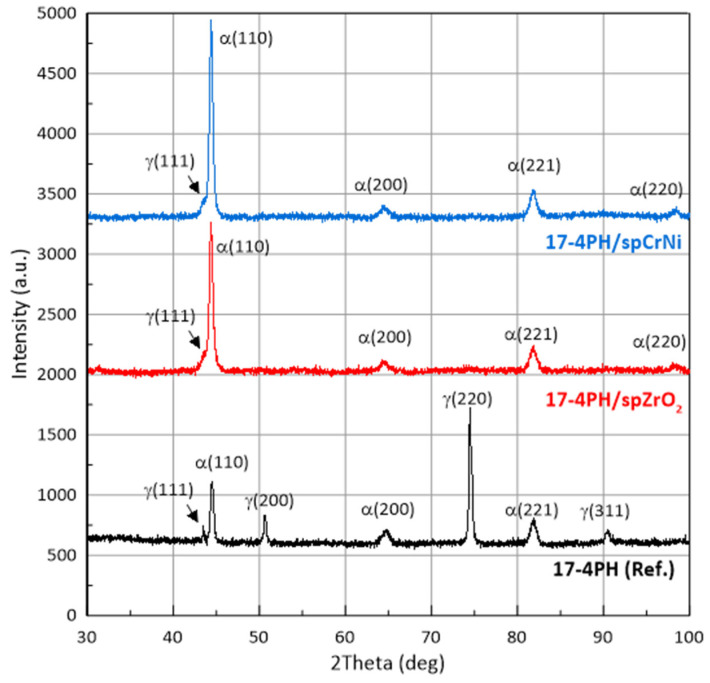
XRD patterns of 17-4PH steel before and after the shot peening process using ZrO_2_ beads and CrNi shots.

The laser sintering process was implemented according to optimized parameters recommended for EOS M280 printers, ensuring a negligible porosity of less than 1%. Moreover, according to the EOS manufacturer, the printing process, which is implemented with a volume rate of 2 mm^3^/s (for 20 μm layers), ensures completely dense components. Finally, the calculated energy density by using Equation (1) was lower than 100 J/mm^2^, which, according to Matilainen et al. [37], is in the optimal range, guaranteeing high efficiency and minimal structure discontinuities. Therefore, the microstructure analysis shown in Figure 3 confirmed no microstructural discontinuities. Moreover, Figure 1 reports a typical surface morphology; the presence of collapsed weld lakes identified only on the “top surface”, which had the character of dimples, increasing surface roughness and no pores or open pores (see Figure 1 and Figure 3), were reported. In order to study the SP process on the phase analysis of 17-4PH steel, XRD-phase analysis were carried out. Figure 4 shows diffraction patterns for 17-4PH steel before and after the shot peening process using ZrO_2_ and CrNi balls. It is easy to see the difference between the XRD profiles for steel before and after the SP process. The steel before treatment showed an austenite-martensite structure, as evidenced by the occurrence of peaks characteristic of austenite located at around 2θ = 43.5°, 50.6°, 75.5° and 90.4°, and the peaks correspond to martensite located at around 2θ = 44.5°, 64.6° and 81.8°. On the other hand, the XRD profiles for steel after the SP process for both types of balls showed the same set of diffraction peaks differing in relative peak intensities. The peak positions corresponded to the martensite structure. Only a relatively low-intensity peak was revealed for 2θ = 43.5°, corresponding to austenite. The individual phases and associated planes were marked at the diffraction peaks in the figure. The disappearance of the austenite peaks in the XRD profiles after the SP process indicates the presence of phase transformation. In addition, a significant increase in the intensity of the (110) coming from the martensite peak, in relation to the (200), (221) and (220) peaks in the profiles for samples after the SP process, indicates that the phase transition had a preferred (110) plane orientation parallel to the sample surface. As a result of the analysis, it was found that the positions of the peaks both before and after the SP process, corresponding to the individual phases, differed by less than 0.12°. However, the FWHM peak analysis showed that the size of martensite grains after the SP process decreased from 22 nm to 15.6 nm and 18.5 nm for the ZrO_2_ and CrNi balls, respectively. On the other hand, the performed Rietveld analysis allowed us to determine the proportion of the number of individual phases. It was calculated that the sample before the SP process consisted of 55% martensite and 42% austenite, and the sample after the SP process consisted of 86% martensite and 14% austenite for the ZrO_2_ beads and 89% martensite and 11% austenite for CrNi balls, respectively. The obtained results are consistent with the results in the literature.

A similar result was obtained by Michela et al. [38]; only the initial samples had a more martensitic structure. This was evident in the absence of distinct peaks from austenite and high hardness after peening. In turn, Wang et al. [39] used a two-stage shot peening using cast iron balls and then ceramic balls with a hardness above 700 HV to determine the effect of the SP process on the phase composition of conventional 17-4PH steel. The research compared the surfaces of the matrix and the laser-hardened layer, where the SP process was carried out with large-diameter spheres (1 mm and 0.1 mm). It was found that SP induced the transformation of austenite to martensite, as evidenced by the disappearance of the austenite peaks in the XRD profile. Moreover, the shift of the martensite peak positions was less than 0.1°. Changes in grain size, micro-strains, and dislocation density were also observed. Similarly, Eskandari et al. [40] confirmed a strain-induced phase transition of austenite to martensite. 

### 3.2. Surface Morphology

**Figure 5:**
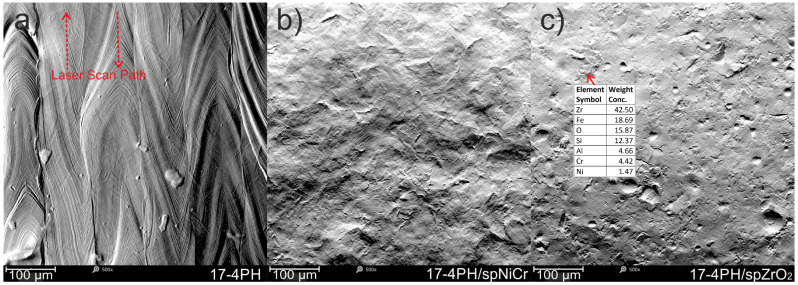
Micrographs of top surfaces of DMLS manufactured 17-4PH samples: (**a**) as-built surface, (**b**) shoot peened using CrNi shots, (**c**) shot peened using ZrO_2_ beads.

SEM imaging in topographic mode allowed for the visualization of the surface both before and after the peening process (Figure 5). The surface visible on the left immediately after fabrication was characterized by overlapping traces of the laser beam passage indicated by red arrows in Figure 5a, above which “splashes” and not surfaces after SP using ceramic beads can be easily distinguished, as smaller irregularities characterize it, while they are full of craters and dents caused by the impact of particles of both spherical and irregular shape. The irregular shape of some depressions is due to the fragmentation of the peening medium. The localization of the bead medium particles in the surface was also observed, in accordance with the Kameyama and Komotori model [41]. The presence of fixed peening medium particles was also confirmed for the ZrO_2_ peened sample using EDS. However, the presence of CrNi steel particles is difficult to confirm using EDS, and this is due to the similar elemental composition to peened 17-4PH steel.

**Figure 6:**
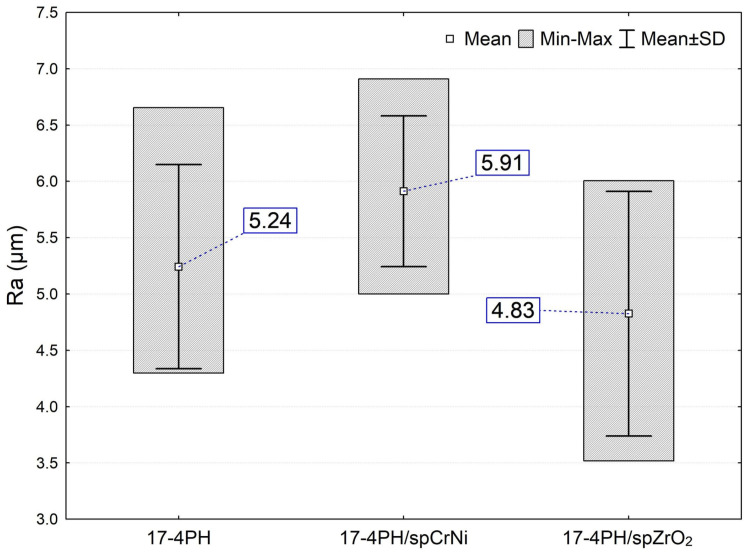
Comparison of mean arithmetic average roughness (*Ra*) of unpeened and shot peened 17-4PH steel.

The results from the roughness (Figure 6) corresponded with the SEM observations of the surfaces. The treatment with ZrO_2_ ceramic beads shows a smoother surface, and the lowest values of the roughness parameter Ra were recorded for these surfaces. In contrast, the treatment of CrNi steels with a shot causes much greater plastic deformation and an increase in roughness even relative to the reference surface. At the same time, it should be bore in mind that this result for the 17-4PH/spCrNi sample was obtained at almost twice the size of the peening medium (vs. ZrO_2_). Comparing roughness with respect to the as-printed surface, there is an increase of 12.8% in the Ra parameter for the surfaces treated with CrNi steels and a decrease of 7.8% for the surfaces treated with ceramic beads. Overall, the DMLS horizontally built material exhibits a significantly rougher texture than wrought steel. According to the research by Mower and Long [42], Sa for laser-sintered surfaces is 3–5 μm. In turn, the magnitude of peened surface roughness depends primarily on the type, size and geometry of the shot, which has already been confirmed in research studies [43,44]. Dorr et al. [43] (for a shot size in the range of 300–600 µm) and also Aymen et al. [44] (for shot size in the range of 125–450 µm) found a proportional increase in surface roughness associated with the use of a larger-size shot.

### 3.3. Hardness of 17-4PH Steel

**Figure 7:**
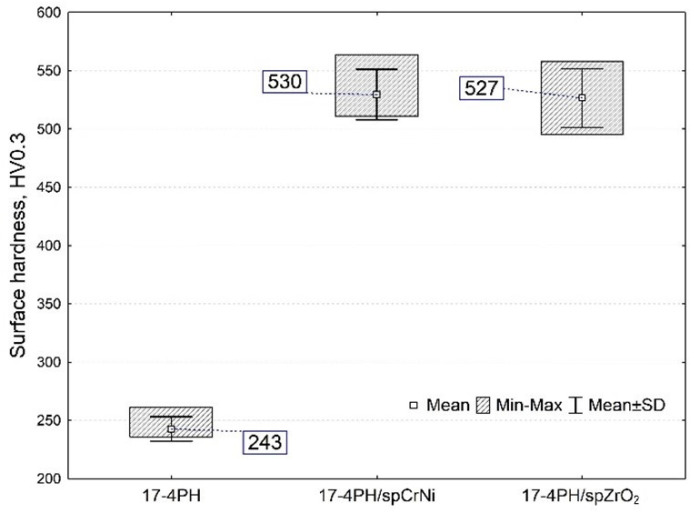
Vickers surface hardness of unpeened and shot peened 17-4PH samples.

The results of the surface hardness tests are shown in Figure 7. An increase in average hardness values was observed for all machined surfaces (by 116.8 ÷ 118.1% on average) compared to the reference samples. Although the highest surface hardness was obtained for the sample 17-4PH/spCrNi, there were no statistically significant differences in hardness for surfaces treated with CrNi shot and ceramics. It is worth recalling that the hardness values for the surfaces treated with ceramic beads were obtained while using a nearly 2.5× smaller shot size. The larger size and weight provide greater impact energy, which in turn, translates into greater hardness. At the same time, the choice of a ceramic with a smaller grain size means that in the same unit of time, a greater number of ceramic balls hit the surface, which results in a higher intensity of the treated surface. In general, the material is strengthened by plastic deformation in the surface layer, at which point the density of dislocations increases. Nevertheless, in the case of ceramic balls, the fragmentation of ceramics occurs during the impacts against the surface. Hard fine particles get buckled (driven in) by successive impact shots, as already documented in Figure 5c. This phenomenon results in a locally significant increase in hardness, which further compensates for the lower impact energy of ceramic beads. 

The average hardness for the untreated samples was 243HV_0.3_, which is comparable to what is claimed by the manufacturer EOS GmbH (230 ± 20 HV). In addition, the average hardness values obtained were similar to the literature data for 17-4PH steels obtained by other additive technologies: Oh et al. [45], 264 HV (laser metal deposition); Chen et al. [46] 265 ± 6 HV (selective laser melting); and Cheruvathur et al. [47] 258 ± 8 HV (laser powder bed fusion). The results showed that shot peening was an efficient cold working method. Analyzing the results from the experiment with the literature, it is apparent that the shot peening for 17-4PH steel produced in AM causes the surface hardness to be significantly higher than in heat treatment, and as the literature data show; for instance, 312 ± 17 HV [47] and 403 HV [45] and 382 ± 10 HV [7]. Such a shot peening treatment will be particularly beneficial when a condition is desired in which the surface of the material is hard and the core is plastic. Wang et al. [48] determined that the surface hardness has a saturation value when peening intensity reaches a specific value from which hardness will no longer increase. They demonstrated that when the peening intensity was increased from 0.3 mmA to 0.6 mmA, the hardness of the hardened 17-4PH steel increased by just about 3–4%.

### 3.4. Tribological Behavior

**Figure 8:**
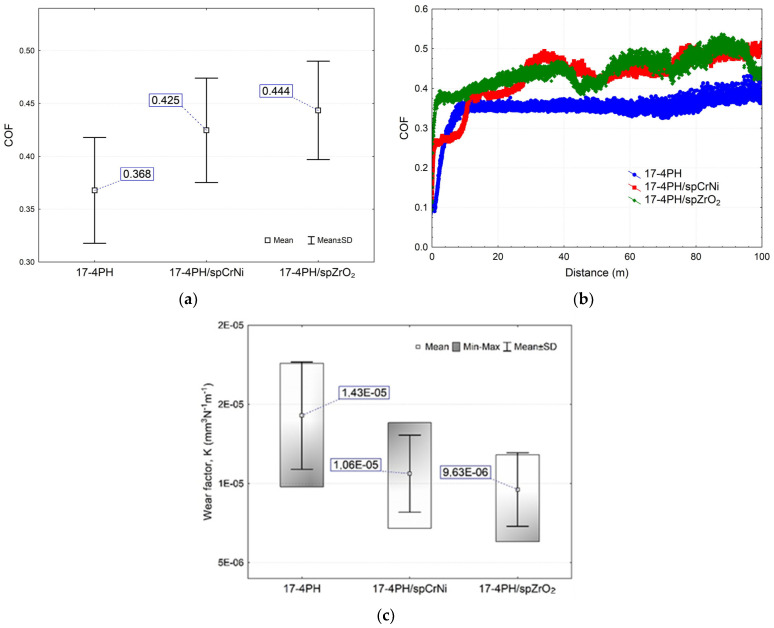
Result of the wear test sliding reported for 17-4PH steel: (**a**) COF mean and standard deviation values; (**b**) COF with regard to distance and (**c**) wear factor.

The results of the wear sliding tests in 0.9% NaCl solution are presented in Figure 8. The observed lower values of the coefficient of friction (COF) for the reference samples are due to the presence of unmelted grains (see Figure 1) after the laser sintering process of metal powder, and the COF itself appears almost stable over the entire route. Slightly higher average COF values were observed for the treated surfaces, with no statistically significant differences. The COF for the surface peened with CrNi shot was slightly lower compared to the surface treated with the ceramic balls. This situation can be explained by the higher surface roughness of the CrNi shot peened surfaces. In turn, higher surface roughness results in tip shear and film lubrication effects at the initial stage of the tribological test. Despite similar COF performances at distances up to about just 30 m (Figure 8b), significantly lower values were recorded for sample 17-4PH/spCrNi compared to 17-4PH/spZrO_2_. In addition, the higher COF values generated for 17-4PH/spZrO_2_ can be explained by the residual hard ceramic particles embedded in the surface layer, which are a natural obstacle to the counterbody [23,41].

Figure 8c shows the test materials’ wear coefficient K results. An increase in wear resistance was observed for all surfaces subjected to shot peening. At the same time, the highest wear resistance was observed for the surfaces modified with ceramic beads, where, in comparison with the reference surface, it is characterized by a nearly 32.7% lower K coefficient. In turn, the modification of the surface with CrN steels shot caused, in relation to the reference surfaces, a decrease in the wear coefficient of 25.9%. 

Comparing the wear results obtained in this work to the tests under technically dry friction conditions reported in the paper [23], one can also see more favorable tribological properties for surfaces treated with ceramic balls. Then, comparatively, for the surface peened with ceramic balls (using the most intensive peening parameters), a decrease in the wear coefficient of nearly 35.6% was obtained. AlMangour and Yang [49] also reported increased wear resistance for shot peened surfaces. The strengthening process was carried out in two steps: by shot peening the surfaces first with aluminum oxide and then with glass beads. An increase in wear resistance was then achieved of approx. ~60.9%. The authors explained that the obtained effect was significantly enhanced through the improved grain refinement effect and the formation of fine-structured surface layers, a stronger surface layer, lower surface roughness and higher microhardness of the DMLS treatment samples. Wang et al. [50] found that the improvement in wear properties of the peened sample results mainly from the harder surface layer, which reduces the degree of plowing and micro-cutting under the lower load and alleviates plastic removal and surface fatigue fracture under the higher load.

**Figure 9:**
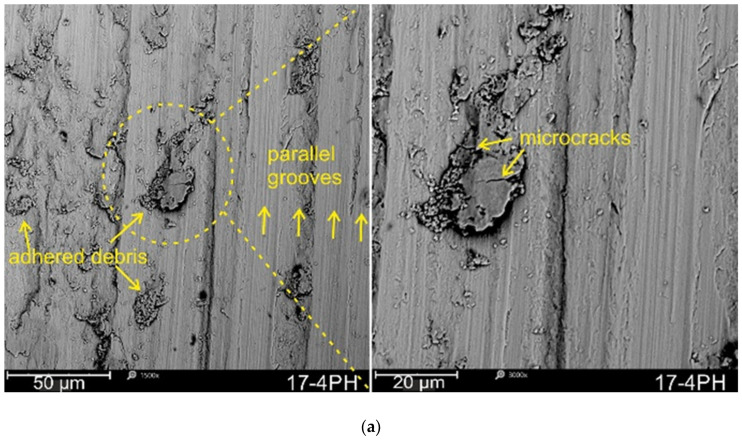

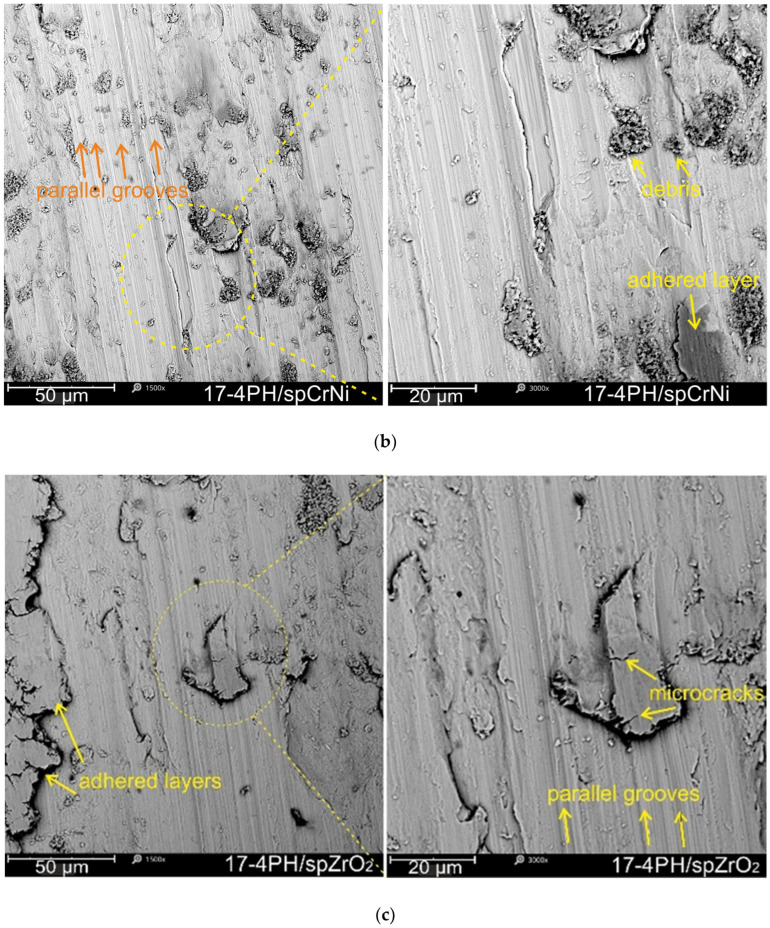
Morphologies of the worn surfaces of additively manufactured 17-4PH steel: (**a**) unpeened (17-4PH), shot peened using (**b**) 17-4PH/spCrNi and (**c**) 17-4PH/spZrO_2_.

Generally, wear is dominated by surface contact; severe surface plastic deformation is expected to have a considerable effect on wear mechanism [49]. SEM images of the worn surfaces are presented in Figure 9. Parallel abrasive and adhesive wear mechanisms predominated on all analyzed surfaces. The abrasive wear mechanism was mainly determined by long parallel grooves along the direction of the counterbody movement. Furthermore, adhesion of secondary wear products transported by the counterbody was observed along the wear marks. In addition, as a result of repeated penetration of the same volume of material by the counterbody, fatigue damage manifested by microcracks occurs. This is in contrast Yang et al. [51], who indicate that fatigue cracks are mainly associated with tests at high loads. Analyzing the wear tracks after tests in 0.9%NaCl and comparing the results with respect to wear tests performed at higher speeds (0.1 m/s) under technically dry friction conditions described in an earlier paper [23], fatigue damage leading to spalling was not observed here. Thus, the observed fatigue mechanism is not as intense as in the case of technically dry friction. 

This is in accordance with AlMangour and Yang [49], in the case of peened samples made with DMLS technology forming a strain-hardened tribolayer that protected the stainless steel from further plowing and spalling, which resulted in a change in the wear mechanism of the strain-hardened tribolayer from abrasive to adhesive. The adhesive and abrasive wear mechanisms for the untreated 17-4PH steel were identified by Esfandiari and Dong [25] during tests in 3.5% NaCl solution. Similar conclusions were drawn by Sanjeev et al. [27], indicating two dominant wear mechanisms, abrasion grooves and smearing, with tribological tests conducted in dry sliding wear conditions.

## 4. Conclusions

This study analyzed the effect of shot peening on tribological behavior in a 0.9% NaCl solution environment of 17-4PH steel produced by direct laser metal sintering (DMLS) technology. The shot peening (SP) was conducted using steel shots (CrNi) and ceramic beads (ZrO_2_). The additively manufactured 17-4PH steel surface roughness, microstructure, XRD phase analysis, Vickers hardness, and the shot peening media’s effect on wear behavior in the wet environment were studied. The wear mechanisms were identified using scanning electron microscopy (SEM). Based on the experimental results, the following was found:

- The peening process conducted using both steel shots and ceramic beads had a favorable effect on the wear resistance of DMLS 17-4PH steel in 0.9%NaCl solution. In particular, ZrO_2_ shot peened surface proved to have a slightly higher wear resistance than the CrNi steel shots. Then, the overall reduction in wear factor (K) compared to the un-peened reference 17-4PH steel was significant and reached 25.9% and 32.7% for the 17-4PH/spCrNi and 17-4PH/spZrO_2_ samples, respectively.
- Shot peening determines the hardness and surface roughness, affecting the tribological behavior of 17-4PH steel. Thus, the usage of CrNi steel shot showed an overall increase of 12.79% Ra mean value compared to the 17-4PH steel that was manufactured. In addition, SP with ZrO_2_ allowed a reduction in the Ra parameter by 7.82%. Therefore, both the SP 17-4PH/spCrNi and 17-4PH/spZrO_2_ samples showed an increased friction coefficient but reduced wear rates than those reported for un-peened 17-4PH.
- The peening process almost doubled the 17-4PH steel hardness of 243 to ~530 HV_0.3_ for the CrNi steel and ZrO_2_ peened samples, respectively. The XRD phase analysis indicated that SP of 17-4PH steel leads to the austenite–martensite phase transition. This resulted in an increase of 118.1% and 116.9% in hardness for CrNi steel and ZrO_2_ peened surfaces, respectively, which was noted. The hardness increase was due to the higher martensite-to austenite content and surface layer grain refinement of shot peened surfaces, both of which contributed to minimalizing the wear rates of the peened 17-4PH/spCrNi and 17-4PH/spZrO_2_ samples.
- Finally, the SEM analysis of wear tracks revealed two wear mechanisms, i.e., abrasive wear determined by parallel grooves and adhesive wear related to the transfer of secondary wear products.

## Figures and Tables

**Table 1:** Chemical composition of shot peening media (wt. %) according to manufacturer (Kuhmichel Abrasiv GmbH).

Ceramic Beads					
ZrO_2_	SiO_2_	Al_2_O_3_	CaO	TiO_2_	Fe_2_O_3_
61.98	27.77	4.57	3.47	0.34	0.14
**CrNi Steel Shots**					
Cr	Ni	Si	Mn	C	Fe
16–20	7–9	1.8–2.2	0.7–1.2	0.05–0.2	Bal.

**Table 2:** Chemical composition (wt. %) of as-fabricated 17-4PH grade steel.

	C	Si	Mn	S	Cr	Mo	Ni	Cu	Co	Nb	V	N	Fe
As-build	0.043	0.694	0.665	0.051	15.18	0.121	4.503	4.734	0.096	0.028	0.054	0.088	Bal.
ASTM A564	<0.07	<0.7	<1.5	-	5–17	<0.6	3–5	3–5	-	5 × C–0.45	-	-	Bal.
EN 10088-1	<0.07	1	<1	-	15–17.5	<0.5	3–5	3–5	-	0.15–0.45	-	-	Bal.

## Data Availability

Data are contained within the article.
